# A retrospective analysis of high sensitivity cardiac troponin-T ranges in non-myocardial infarction emergency department visits

**DOI:** 10.1186/s12872-021-02089-0

**Published:** 2021-06-07

**Authors:** Nathan Kong, Rhys F. M. Chua, Stephanie A. Besser, Louise Heelan, Sandeep Nathan, Thomas F. Spiegel, Xander M. R. van Wijk, Corey E. Tabit

**Affiliations:** 1 Department of Medicine, The University of Chicago, Chicago, IL USA; 2 Department of Medicine, Section of Cardiology, The University of Chicago, 5841 South Maryland Avenue, MC6080, Chicago, IL 60637 USA; 3 Department of Data Science and Analytics, The University of Chicago Medicine, Chicago, IL USA; 4 Department of Medicine, Section of Emergency Medicine, The University of Chicago, Chicago, IL USA; 5 Department of Pathology, The University of Chicago, Chicago, IL USA

**Keywords:** Cardiac troponin, Acute myocardial infarction, Chronic kidney disease, Emergency Department

## Abstract

**Introduction:**

Current evidence suggests that high sensitivity cardiac troponin-T (hs-cTnT) values differ based on sex, race, age, and kidney function. However, most studies examining the relationship of hs-cTnT and these individual factors are in healthy participants, leading to difficulty in interpreting hs-cTnT values in the Emergency Department (ED) setting. We seek to examine the relationship between hs-cTnT values and sex, race, age, and kidney function in a contemporary, urban academic setting.

**Methods:**

ED visits from June 2018 through April 2019 with at least 1 hs-cTnT and no diagnosis of acute myocardial infarction (AMI) at an academic medical center in the south side of Chicago were retrospectively analyzed. Median hs-cTnT values were stratified by sex (male or female), race (African American or Caucasian), age, estimated glomerular filtration rate (eGFR), and stage of chronic kidney disease.

**Results:**

9679 encounters, representing 7989 distinct patients, were included for analysis (age 58 ± 18 years, 59% female, 85% black). Males had significantly higher median hs-cTnT values than females (16 [8–34] vs. 9 [6–22] ng/L, *p* < 0.001), African Americans had a significantly lower median value than Caucasians (10 [6–24] vs. 15 [6–29] ng/L, *p* < 0.001), and those with atrial fibrillation (27 [16–48] vs. 9 [6–19] ng/L, *p* < 0.001) and heart failure (28 [14–48] vs. 8 [6–15] ng/L, *p* < 0.001) had higher median values than those without. Median hs-cTnT values increased significantly with increased age and decreased eGFR. All relationships continued to be significant even after multivariable regression of sex, age, race, eGFR, presence of atrial fibrillation, and presence of heart failure (*p* < 0.01).

**Conclusions:**

Analysis of hs-cTnT in non-AMI patients during ED encounters showed that males have higher values than females, African Americans have lower values than Caucasians, those with atrial fibrillation and heart failure have higher values than those without, and that older age and lower eGFR were associated with higher median values.

## Introduction

Since the approval of the high-sensitivity cardiac troponin-T (hs-cTnT) assay by the US Food and Drug Administration in 2017 for clinical care, there has been renewed interest in determining blood concentrations in individuals without acute myocardial infarctions (AMI) [1, 2]. Studies have suggested that hs-cTnT levels vary by sex, race, age, and degree of kidney function [3–6]. However, many of these studies were conducted in healthy participants without other major comorbidities. As a result, clinicians often have difficulty interpreting hs-cTnT values in the setting of individual patient factors, such as older patients with chronic kidney disease (CKD) [7, 8]. Herein, we describe the ranges of hs-cTnT in Emergency Department (ED) visits across sex, race, age, estimated glomerular filtration rate (eGFR), and presence of heart failure or atrial fibrillation in patients without AMI during usual clinical care.

## Materials and methods

**Fig. 1:**
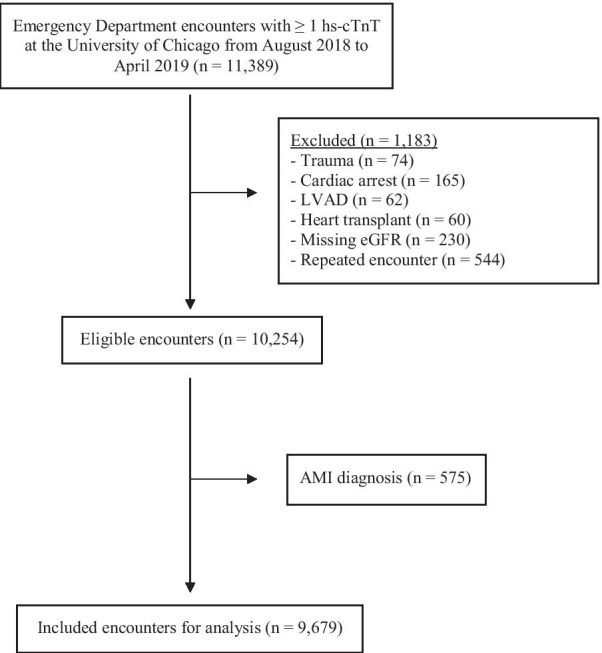
Flowsheet of patient inclusion

Hs-cTnT values from all ED encounters from August 2018 through April 2019 at an academic medical center in the south side of Chicago were retrospectively analyzed. Patients with repeated encounters within 14 days, trauma, cardiac arrest, left ventricular assist devices (LVAD), history of heart transplant, patients without an eGFR value, and patients diagnosed with AMI during the encounter were excluded from analysis (Fig. 1). Sex and race were self-reported at the time of registration. All hs-cTnT tests were performed using the Roche Diagnostics Troponin T Gen 5 assay. Initial verification of the assay was performed on two separate Roche Cobas e602 analyzers. Further information regarding the analytical precision of the assay at various concentrations using three patient pools is provided in Additional file 1: sFigure1. The decision for hs-cTnT testing was based on individual clinician judgement using clinical history, the patient’s past medical history, and other clinical context. For each ED encounter, a single hs-cTnT was calculated as the median of all collected tests for the encounter. Unquantifiable hs-cTnT values were assigned a value of 6 ng/L (the limit of quantification). AMI diagnoses were determined by an associated International Classification of Disease version 10 (ICD-10) diagnosis of acute myocardial infarction (I21–I24) during the encounter. eGFR was calculated using the Modification of Diet in Renal Disease (MDRD) as described elsewhere [9]. End-stage renal disease (ESRD) was defined using ICD-10 codes and current procedural terminology (CPT) codes for dialysis. CKD stages was determined by eGFR cutoffs based on the National Kidney Foundation’s 2002 guidelines on the classification of CKD [10].

At our center, the protocol described by Vigen et al. [7] is used to rule out/in AMI in the ED. Briefly, hs-cTnT is drawn upon arrival in the ED; if the hs-cTnT value is < 6 ng/L and the symptom duration is ≥ 3 h, the patient rules out for AMI. If the initial hs-cTnT value is ≥ 6 and < 52 ng/L, or if the initial hs-cTnT value is < 6 ng/L and the symptom duration is < 3 h, a second hs-cTnT is drawn 1 h later. If this second hs-cTnT value is < 12 ng/L and the change from the initial hs-cTnT to the second hs-cTnT is < 3 ng/L, the patient rules out for AMI. If the second hs-cTnT value is ≥ 12 and < 52 ng/L and the change from the initial hs-cTnT to the second hs-cTnT is ≥ 5 ng/L, the result is considered abnormal and further clinical evaluation for likely AMI is undertaken by a cardiologist. If the second hs-cTnT value is ≥ 12 and < 52 ng/L and the change from the initial hs-cTnT to the second hs-cTnT is < 5 ng/L, or if the second hs-cTnT value is < 12ng/L but the change from the initial hs-cTnT to the second hs-cTnT is ≥ 3 ng/L, a third hs-cTnT is drawn 2 h later (3 h after the initial draw). If the change from the initial hs-cTnT to the third hs-cTnT is < 7 ng/L, the patient rules out for AMI. If the change is ≥ 7 ng/L, the result is considered abnormal and further clinical evaluation for likely AMI is undertaken by a cardiologist. Finally, any hs-cTnT value ≥ 52 ng/L is considered abnormal and further clinical evaluation for likely AMI is undertaken by a cardiologist. The cutoff values for hs-cTnT in this protocol are the same for both male and female patients. For patients considered likely for AMI, the cardiologist then uses a combination of electrocardiographic and laboratory data and findings on history and physical examination to make or exclude the clinical diagnosis of AMI.

Continuous baseline clinical variables were expressed as means ± standard deviations or medians with interquartile ranges (IQR) if non-normally distributed. Categorical baseline clinical variables were expressed as counts and percentages. Missing data was excluded. Outlier data was removed prior to percentile (%tile) analysis (Table 1). Outliers were considered 1.5 times the IQR. Median hs-cTnT values were compared using Mann-Whitney U test with a two-tailed p-value < 0.05 being statistically significant for all analyses. Each point on the scatter plots represents the median hs-cTnT value of all encounters within the specified x-value. Spearman’s rank correlation coefficients were used to determine the associations between hs-cTnT to age and eGFR. All analyses and visualizations were performed with ‘Tidyverse’, ‘data.table’, and ‘sjPlot’ packages in R 4.0.2 (R Core Team 2020). The University of Chicago Institutional Review Board (IRB) reviewed and approved the study (IRB 17-0324). All methods were carried out in accordance with relevant guidelines and regulations.

**Table 1:** hs-cTnT values (in ng/L) at the 25th, 50th, 75th, and 99th percentiles stratified by sex, race, age group, CKD stage, history of atrial fibrillation, and history of heart failure. Results are aggregated at the encounter level

	n	25th %tile	50th %tile	75th %tile	99th %tile
Sex					
Female	5099	6	8	16	48
Male	3487	8	15	29	81
Race					
Black	7414	6	10	21	61
White	854	6	15	28	73
Age group					
< 50	2427	6	6	7	18
50–64	2789	6	10	19	54
≥ 65	3238	11	20	34	86
CKD stage					
No CKD	3004	6	6	9	21
Stage 2	3009	6	10	17	41
Stage 3	1725	13	22	35	80
Stage 4	390	28	44	70	168
Stage 5	121	43	66	110	225
ESRD	551	62	96	152	312
Atrial fibrillation					
No	7688	6	9	19	54
Yes	966	16	27	48	122
Heart failure					
No	6554	6	8	15	41
Yes	2060	14	28	48	126

%tile, percentile

## Results

**Fig. 2:**
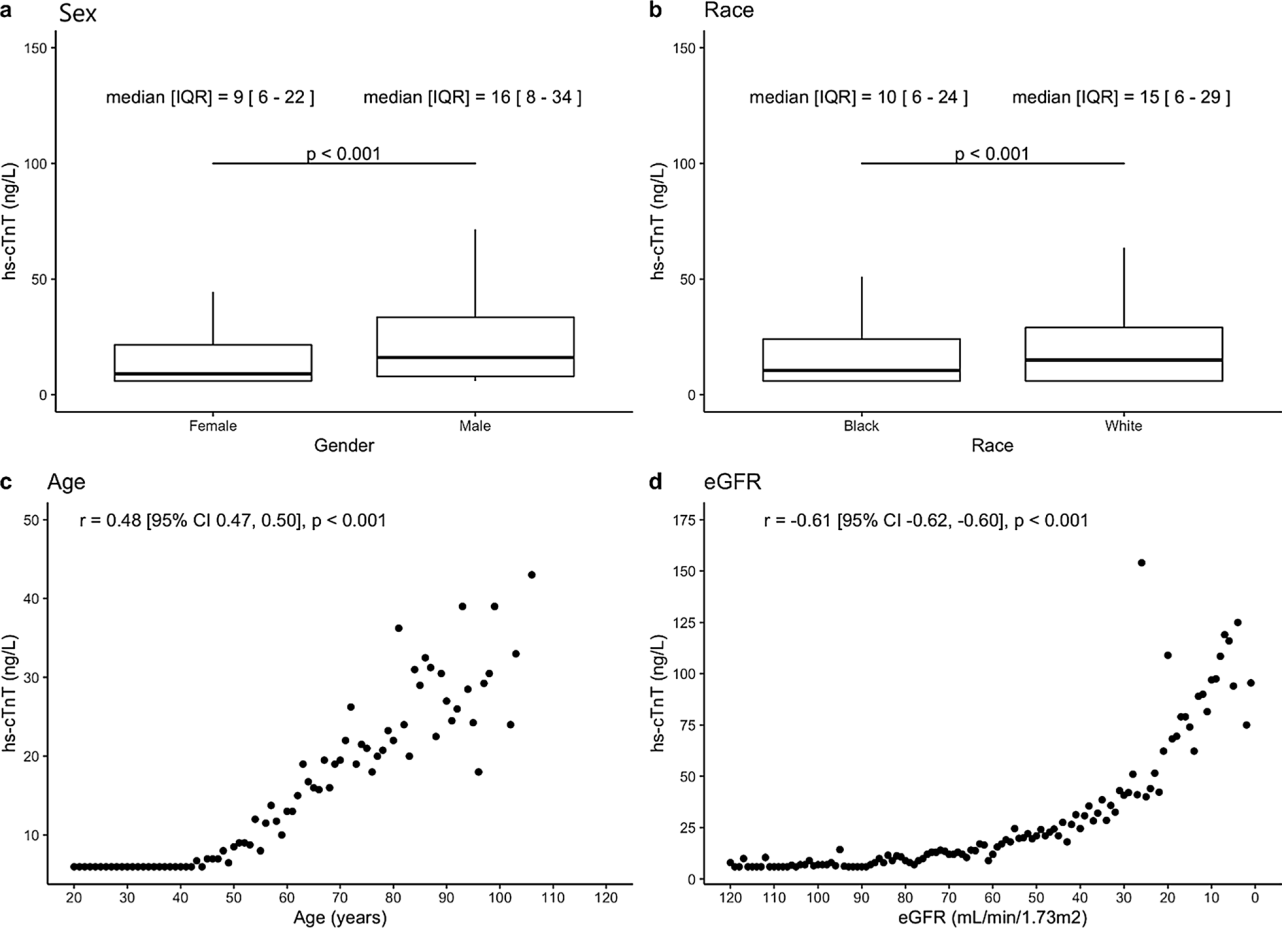
Boxplots and scatter plots of hs-cTnT relationship by sex, race, age, and eGFR. For **c**, **d** each dot represents the median hs-cTnT value for all encounters with that age or eGFR, respectively

11,389 individual ED encounters were collected. 9679 encounters, representing 7989 distinct patients, were included for analysis (Fig. 1). Mean age was 57.8 ± 17.6 years, 59.3% were female, 85.0% were Black (Table 2). The median hs-cTnT across all included encounters was 12.0 [IQR 6.0–29.5] ng/L. 3,061 (31.6%) encounters had median hs-cTnT values at or below the limit of quantification. Males had significantly higher median hs-cTnT values than females (16 [8–34] vs. 9 [6–22] ng/L, *p* < 0.001, Fig. 2a), African Americans had a significantly lower median value than Caucasians (10 [6–24] vs. 15 [6–29] ng/L, *p* < 0.001, Fig. 2b), and those with atrial fibrillation (27 [16–48] vs. 9 [6–19] ng/L, *p* < 0.001) and heart failure (28 [14–48] vs. 8 [6–15] ng/L,  *p* < 0.001) had higher median values than those without (Table 1). All relationships continued to be significant even after multivariable regression of sex, age, race, eGFR, presence of atrial fibrillation, and presence of heart failure (*p* < 0.01).

**Table 2:** Baseline demographics by sex, aggregated on the patient level. For repeated encounters, only the first patient encounter was used. Categorical variables expressed as count (percent). Normally distributed continuous variables expressed as mean (standard deviation), non-normally distributed continuous variables expressed as mean [inter-quartile range]

	Overall (n = 7989)	Female (n = 4734)	Male (n = 3255)	*p* value*
Age (years)	57.8 (17.6)	57.9 (18.3)	57.8 (16.6)	0.795
Black	6789 (85)	4141 (87)	2648 (81)	< 0.001
BMI (kg/m^2^)	28.0 [23.0, 34.0]	29.0 [24.0, 36.0]	27.0 [23.0, 31.0]	< 0.001
eGFR (mL/min/1.73 m^2^)	67.0 [47.0, 85.0]	67.0 [47.0, 84.0]	67.0 [47.0, 86.0]	0.484
Systolic blood pressure (mmHg)	140.0 [122.0, 157.0]	141.0 [123.0, 158.0]	137.0 [120.0, 155.0]	< 0.001
Diastolic blood pressure (mmHg)	82.0 (19.0)	81.4 (18.6)	82.8 (19.5)	0.001
NT-proBNP (pg/mL)	418.0 [87.0, 2485.0]	321.0 [79.0, 1868.5]	642.5 [106.2, 3426.0]	< 0.001
Sodium (mEq/L)	140.0 [138.0, 142.0]	140.0 [138.0, 142.0]	140.0 [137.0, 142.0]	< 0.001
Hemoglobin (g/dL)	12.5 [10.9, 13.8]	12.2 [10.8, 13.3]	13.1 [11.3, 14.5]	< 0.001
Low-density Lipoprotein (mg/dL)	80.0 [60.0, 109.0]	83.5 [63.0, 115.0]	76.0 [55.0, 101.0]	< 0.001
Total cholesterol (mg/dL)	156.2 [128.2, 190.4]	163.2 [134.8, 198.8]	146.6 [120.2, 174.2]	< 0.001
Diabetes mellitus	2007 (25)	1181 (25)	826 (25)	0.683
Hypertension	4613 (58)	2703 (57)	1910 (59)	0.167
Coronary Artery Disease	1095 (14)	526 (11)	569 (17)	< 0.001
Heart failure	1645 (21)	890 (19)	755 (23)	< 0.001
Atrial fibrillation	785 (10)	367 (8)	418 (13)	< 0.001

*p value comparisons are between females and males only

Patients older than or equal to 65 years had significantly higher median troponin values (20 [11–34] ng/L) compared with those between 50 and 64 years (10 [6–19] ng/L, *p* < 0.001) and patients less than 50 years (6 [6–7] ng/L, *p* < 0.001). Median hs-cTnT were at the level of detection until age 40 at which point a direct linear relationship existed between age and median troponin value (Fig. 2c, r = 0.48 [95% CI 0.47–0.50], *p* < 0.001). This relationship remained even during sub-group analysis of encounters where eGFR was ≥ 90 mL/min/1.73 m^2^ (r = 0.50 [95% CI 0.47–0.53], *p* < 0.001, Fig. 2).

The median hs-cTnT value of patients without CKD was 6 [6–9] ng/L which was significantly lower when compared to stage 2 CKD (10 [6–17] ng/L), stage 3 CKD (22 [13–35] ng/L), stage 4 CKD (44 [28–70] ng/L), stage 5 CKD (66 [43–110] ng/L), and in patients with ESRD (96 [62–152] ng/L). An inverse exponential relationship existed between eGFR and median hs-cTnT (Fig. 2d, r = − 0.61 [95% CI − 0.62 to -0.60], *p* < 0.001).

hs-cTnT values at the 25th, 50th, 75th, and 99th percentiles for age, sex, race, and CKD subgroups are presented in Tables 1 and 3.

**Table 3:** hs-cTnT values (in ng/L) at the 25th, 50th, 75th, and 99th percentiles stratified by multiple subgroups. Results are reported aggregated at the encounter level

Sex	Race	Age group	eGFR	n	25th %tile	50th %tile	75th %tile	99th %tile
Female	B	< 50	≥ 90	887	6	6	6	6
			60–89	319	6	6	6	8
			< 60	196	11	28	67	184
Female	B	50–64	≥ 90	465	6	6	7	14
			60–89	540	6	7	11	24
			< 60	414	14	26	47	99
Female	B	≥ 65	≥ 90	238	8	12	20	50
			60–89	641	8	12	19	47
			< 60	805	14	25	43	112
Female	W	< 50	≥ 90	41	6	6	6	6
			60–89	37	6	6	6	6
			< 60	15	6	18	25	56
Female	W	50–64	≥ 90	15	6	9	16	30
			60–89	51	6	6	10	23
			< 60	27	8	14	28	53
Female	W	≥ 65	≥ 90	33	10	12	21	124
			60–89	89	9	15	23	56
			< 60	107	19	28	52	104
Male	B	< 50	≥ 90	441	6	6	8	15
			60–89	232	6	9	15	33
			< 60	152	34	69	139	306
Male	B	50–64	≥ 90	420	6	10	16	31
			60–89	383	8	13	20	49
			< 60	319	21	43	82	208
Male	B	≥ 65	≥ 90	212	11	16	25	48
			60–89	376	12	19	32	65
			< 60	454	27	46	84	204
Male	W	< 50	≥ 90	44	6	6	7	22
			60–89	30	6	6	16	28
			< 60	10	9	28	49	66
Male	W	50–64	≥ 90	30	8	13	20	46
			60–89	57	8	13	20	55
			< 60	37	24	45	62	519
Male	W	≥ 65	≥ 90	50	13	19	34	101
			60–89	74	12	17	27	43
			< 60	102	23	37	63	116

B, Black; W, White, %tile, percentile

## Discussion

ED encounters from a large, tertiary, academic medical center showed that hs-cTnT values differed considerably by sex, race, age, CKD stage, and presence of atrial fibrillation and heart failure in patients without AMI. Our results from routine clinical care of non-AMI patients support data from healthy participants indicating that males have higher hs-cTnT values than females [2, 4, 5]. Our results indicate that these relationships exist across race and eGFR strata. Of note, the manufacturer-recommended 99th percentiles are 19 ng/L for a US population, 14ng/L for US females, and 22 ng/L for US males. There are no manufacturer recommendations for 99th percentiles by race, age, or CKD stage.

It has also been shown in healthy cohorts that older individuals have higher troponin values than younger individuals [5]. It appears from Fig. 2c that this relationship exists from age 45–85. Age and median hs-cTnT does not appear to have a relationship at the extremes of age (Fig. 2c). While eGFR is also related to age, when controlling for eGFR by analyzing only those without CKD, a similar relationship between age and median hs-cTnT was observed. The pathophysiology behind this phenomenon is unclear but it is likely a combination of both physiology and pathology, such as higher rates of co-morbidities [4].

The relationship between race and hs-cTnT is even less clear in the literature. Data from healthy participants have shown multiple different relationships with some studies suggesting no difference or lower hs-cTnT levels in African Americans compared with Caucasians whereas others have seen increased levels of hs-cTnT in African Americans [2, 5]. A sub-study of the Dallas Heart Study (DHS), a multiethnic, population cohort of residents in Dallas County, showed that black women had higher hs-cTnT levels but after adjustment this relationship disappeared [11]. Our results from an unprecedented percentage of African Americans (85%) show that hs-cTnT levels are lower in African American patients than Caucasian patients. The literature is sparse as to the biologic mechanism by which race would play a role in hs-cTnT concentrations. Interestingly, whites in our study had higher rates of atrial fibrillation and coronary artery disease but lower rates of diabetes, hypertension, and kidney disease (Additional file 1: sTable 2). It is possible that there is no true physiologic reason for these differences and rather a confounder remains unaccounted. However, we believe the racial differences remain an important finding particularly given the disparities in AMI diagnosis between races and should be further studied.

Previous studies have shown that hs-cTnT is inversely related to eGFR [12]. While the exact pathophysiology is unknown, it is thought to be a combination of kidney disease-related subclinical cardiac damage with a minor contribution of impaired renal clearance of cTnT fragments by the kidney membrane [13]. There is still unclear guidance on how to interpret hs-cTnT values in the setting of kidney disease. Our results from a large number of hs-cTnT values in patients without AMI during routine clinical care indicate an inversely exponential relationship between eGFR and median hs-cTnT. When broken down by stage of CKD, it appears that the median hs-cTnT of the CKD stage group doubles until CKD5 and ESRD where a 50% increase is observed for each progressive CKD stage (Table 1).

Our study has a number of notable strengths. First, we include a large number of patients in the setting of routine clinical care. We believe this is a more clinically relevant population to study as opposed to many other studies which have attempted to characterize troponin values in the setting of healthy cohorts. Second, our study included 85% African Americans. These numbers allow for unprecedented insight into an often under-represented minority in clinical research. Lastly, because of the heterogenous nature of our patient population, we were able to characterize a large range of ages and eGFR including over 600 patients who were on chronic dialysis.

Several limitations should be noted. We recognize exclusion based on ICD codes for AMI is likely incomplete due to mis-classification. In addition, while we excluded patients with AMI, there are patients in our study with conditions that are also known to cause elevations in cardiac troponins (i.e.: pulmonary embolism, myocarditis). As our study described hs-cTnT values in a diverse ED population, it was beneficial to include all conditions except AMI where hs-cTnT change and rate of rise are important diagnostic information. Second, the 99th percentile values in certain sub-groups should be examined with caution as some of the Caucasian groups have low numbers (i.e.: less than 100). Also, the staging of chronic kidney disease based on a single creatinine value taken at the time of acute illness is likely to overestimate the severity of kidney disease. However, data in the context of a single eGFR is more generalizable as clinicians are often required to interpret troponin levels with a single data point.

We chose to use a median value of hs-cTnT across the encounter to give a global representation of hs-cTnT within an encounter. Mean values would be skewed due to the non-normal distribution of hs-cTnT and the maximum values would not be as useful to clinicians in the context of non-AMI patients. While serial testing of hs-cTnT may affect median values, we do not think that this is a major limitation as we excluded patients with AMI and thus most patients would have values that remained stable. It is also worth mentioning that the diagnosis of AMI requires a combination of clinical context and changes in hs-cTnT values over time. Our institution utilizes the rule-out method suggested by Vigen et al. prior to an expert clinician (both a hospital medicine clinician and/or a licensed cardiologist) making the diagnosis of AMI(7). Despite the exclusion of all patients with a diagnosis of AMI from analysis, residual confounders likely remain. Specifically, some patients that were included may have had AMI even though they were not classified as such and some excluded patients did not have AMI. However, our study is focused more on the expected ranges of hs-cTnT values observed in a standard ED population as opposed to making a diagnosis of AMI. It is also worth noting that we excluded outliers in our main analysis as our dataset included extreme values (i.e. > 10,000 ng/L), beyond the measuring limit of our assay. Since it was not possible to determine if these values were due to error, we felt it most appropriate to exclude them. Additionally, hs-cTnT values at the extreme limits are difficult to interpret without clinical context. However, we have also provided data on percentiles without outliers excluded in Additional file 1: sTable 3 and Additional file 1: sTable 4. Finally, it is very important to further stress and acknowledge that myocardial injury is common in hospitalized patients. The population in this study is likely to include substantial numbers of patients with cardiac ischemia in the context of multiple systemic and cardiac conditions other than AMI. This will have implications on the presented results, and consequently the 99th percentile values reported here should not be considered as informative for the clinical diagnosis of AMI in any of the subgroups reported.

Analysis of hs-cTnT in non-AMI patients during routine clinical care at a large, diverse tertiary ED in a contemporary US population showed that males have higher hs-cTnT values than females, African Americans have lower values than Caucasians, older age was associated with higher values, and lower eGFR was associated with higher values. These results suggest that these patient specific factors should be incorporated into the interpretation of hs-cTnT values.

## Supplementary Information

**Additional file 1: sFigure 1**. Imprecision (% CV) at various levels of hs-cTnT during initial verification. Three patient pools are measured on two Roche Cobas e602 analyzers once per day for 20 days (40 points per pool). 10% CV is estimated at 11 ng/L cTnT. **sTable 1**. The top 10 reasons for emergency department visits by number of encounters. **sTable 2**. Baseline demographics by race, aggregated on the patient level. For repeated encounters, only the first patient encounter was used. Categorical variables expressed as count (percent). Normally distributed continuous variables expressed as mean (standard deviation), non-normally distributed continuous variables expressed as mean [inter-quartile range]. **sFigure 2**. Scatter plot of BMI and high sensitivity cardiac troponin T. Negative weak correlation. sFigure 3. Scatter plot of age with only encounters where eGFR ≥ 90 mL/min/1.73 m^2^. **sTable 3**. hs-cTnT values at the 25th, 50th, 75th, and 99th percentiles stratified by sex, race, age group, CKD stage, history of atrial fibrillation, history of heart failure without removing outliers. Results are aggregated at the encounter level. **sTable 4**. hs-cTnT values at the 25th, 50th, 75th, and 99th percentiles stratified by multiple subgroups without outliers removed. Results are reported aggregated at the encounter level.

## Data Availability

A de-identified dataset generated and used during the current study can be made available from the corresponding author on request and with approval from the IRB at the University of Chicago.

## Abbreviations

hs-cTnT: High-sensitivity cardiac troponin-T
AMI: Acute myocardial infarctions
ED: Emergency Department
eGFR: Estimated glomerular filtration rate
MDRD: Modification of Diet in Renal Disease
ESRD: End-stage renal disease
IQR: Interquartile ranges
CPT: Current procedural terminology
CKD: Chronic kidney disease
LVAD: Left ventricular assist devices
IRB: Institutional Review Board
