# Supplementary material for: A retrospective analysis of high sensitivity cardiac troponin-T ranges in non-myocardial infarction emergency department visits
Source: BMC Cardiovasc Disord. 2021 Jun 7;21:283. doi: 10.1186/s12872-021-02089-0 (PMC8186234; doi:10.1186/s12872-021-02089-0)
Supplement: Supplementary file 1 — Additional file 1: sFigure 1. Imprecision (% CV) at various levels of hs-cTnT during initial verification. Three patient pools are measured on two Roche Cobas e602 analyzers once per day for 20 days (40 points per pool). 10% CV is estimated at 11 ng/L cTnT. sTable 1. The top 10 reasons for emergency department visits by number of encounters. sTable 2. Baseline demographics by race, aggregated on the patient level. For repeated encounters, only the first patient encounter was used. Categorical variables expressed as count (percent). Normally distributed continuous variables expressed as mean (standard deviation), non-normally distributed continuous variables expressed as mean [inter-quartile range]. sFigure 2. Scatter plot of BMI and high sensitivity cardiac troponin T. Negative weak correlation. sFigure 3. Scatter plot of age with only encounters where eGFR ≥ 90 mL/min/1.73 m2. sTable 3. hs-cTnT values at the 25th, 50th, 75th, and 99th percentiles stratified by sex, race, age group, CKD stage, history of atrial fibrillation, history of heart failure without removing outliers. Results are aggregated at the encounter level. sTable 4. hs-cTnT values at the 25th, 50th, 75th, and 99th percentiles stratified by multiple subgroups without outliers removed. Results are reported aggregated at the encounter level. [file 12872_2021_2089_MOESM1_ESM.pdf]

A Retrospective Analysis of High Sensitivity Cardiac Troponin-T Ranges in Non-Myocardial Infarction Emergency Department Visits

Nathan Kong[1], Rhys FM Chua[2], Stephanie A Besser[2], Louise Heelan[3], Sandeep Nathan[2], Thomas F. Spiegel[4], Xander MR van Wijk[5], Corey E Tabit[2]

Supplemental

sFigure 1. Imprecision (% CV) at various levels of hs-cTnT during initial verification. Three patient pools are measured on two Roche Cobas e602 analyzers once per day for 20 days (40 points per pool). 10% CV is estimated at 11 ng/L cTnT.

sTable 1. The top 10 reasons for emergency department visits by number of encounters.

sTable 2. Baseline demographics by race, aggregated on the patient level. For repeated encounters, only the first patient encounter was used. Categorical variables expressed as count (percent). Normally distributed continuous variables expressed as mean (standard deviation), non-normally distributed continuous variables expressed as mean [inter-quartile range].

sFigure 2. Scatter plot of BMI and high sensitivity cardiac troponin T. Negative weak correlation.

sFigure 3. Scatter plot of age with only encounters where eGFR ≥ 90 mL/min/1.73m^2

sTable 3. hs-cTnT values at the 25th, 50th, 75th, and 99th percentiles stratified by sex, race, age group, CKD stage, history of atrial fibrillation, history of heart failure without removing outliers. Results are aggregated at the encounter level.

sTable 4. hs-cTnT values at the 25th, 50th, 75th, and 99th percentiles stratified by multiple subgroups without outliers removed. Results are reported aggregated at the encounter level.

Supplemental Figure 1. Imprecision (% CV) at various levels of hs-cTnT during initial verification. Three patient pools are measured on two Roche Cobas e602 analyzers once per day for 20 days (40 points per pool). 10% CV is estimated at 11 ng/L cTnT.

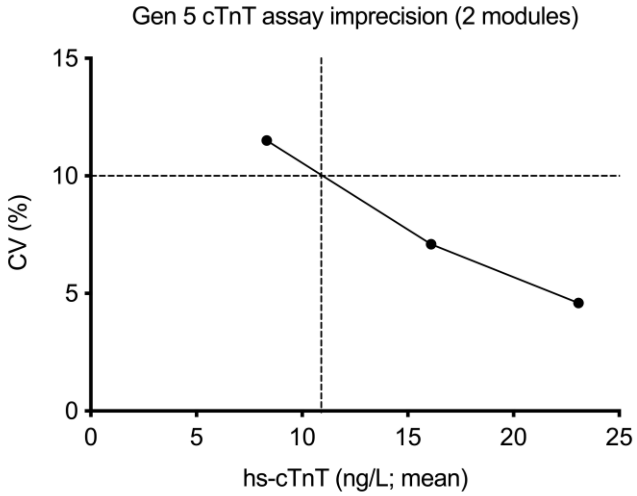

Supplemental Table 1. The top 10 reasons for emergency department visits by number of encounters

Reason for ED Visit	Number of Encounters
Chest pain	1,766
Hypertension	908
Shortness of breath	859
Pain, not chest	784
Syncope, Near Syncope	378
Sepsis	346
Headache	150
Palpitations	117
Pulmonary embolism	112
Cough	92

Supplemental Table 2. Baseline demographics by white versus black race, aggregated on the patient level. For repeated encounters, only the first patient encounter was used. For this table only, all other races excluded. Categorical variables expressed as count (percent). Normally distributed continuous variables expressed as mean (standard deviation), non-normally distributed continuous variables expressed as mean [inter-quartile range].

	White (n = 846)	Black (n = 6,789)	p-value
**Age (yrs)**	62.1 (17.7)	57.5 (17.5)	<0.001
**Male**	426 (50)	2648 (39)	<0.001
**BMI (kg/m2)**	26.0 [23.0, 30.0]	28.0 [24.0, 34.0]	<0.001
**eGFR (mL/min/1.73m2)**	71.0 [51.0, 91.0]	65.0 [46.0, 83.0]	<0.001
**Systolic blood pressure (mmHg)**	130.0 [114.0, 147.0]	141.0 [123.0, 158.0]	<0.001
**Diastolic blood pressure (mmHg)**	75.9 (17.5)	82.8 (19.0)	<0.001
**NT-proBNP (pg/mL)**	661.5 [148.8, 2502.5]	383.5 [83.0, 2469.5]	0.001
**Sodium (mEq/L)**	139.0 [136.0, 141.0]	140.0 [138.0, 142.0]	<0.001
**Hemoglobin (g/dL)**	12.7 [10.8, 14.2]	12.4 [10.9, 13.7]	0.002
**Low-density Lipoprotein (mg/dL)**	85.0 [64.5, 107.0]	80.0 [59.0, 110.0]	0.216
**Total cholesterol (mg/dL)**	166.4 [138.5, 188.9]	154.8 [126.6, 191.2]	0.019
**Diabetes mellitus**	163 (19)	1752 (26)	<0.001
**Hypertension**	410 (48)	4047 (60)	<0.001
**Coronary Artery Disease**	149 (18)	902 (13)	0.001
**Heart failure**	156 (18)	1432 (21)	0.08
**Atrial fibrillation**	160 (19)	590 (9)	<0.001

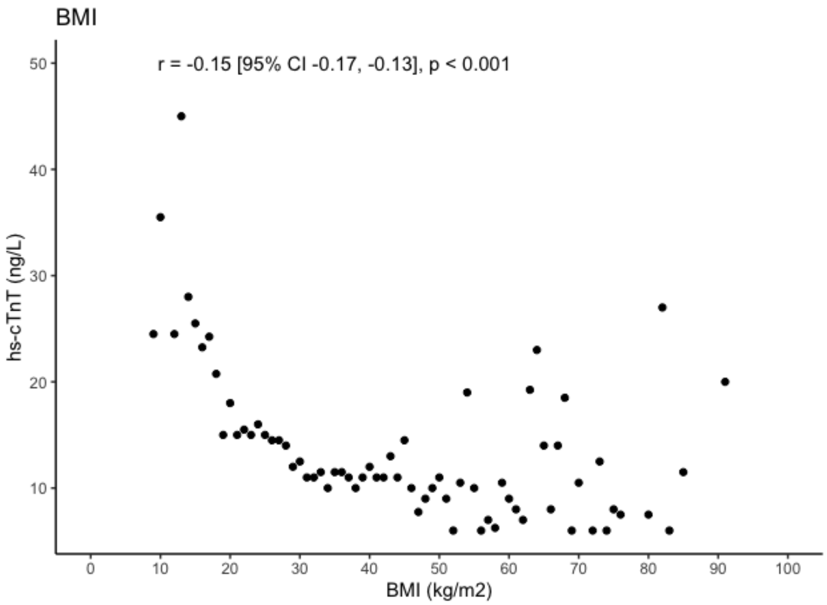

Supplemental Figure 1. Scatter plot of BMI and high sensitivity cardiac troponin T. Negative weak correlation.

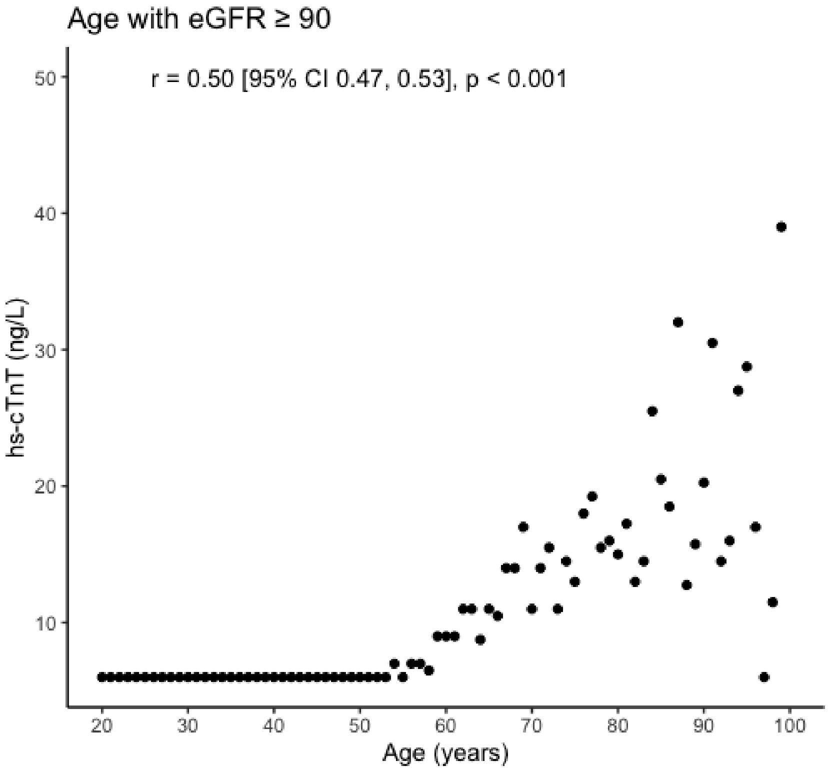

Supplemental Figure 2. Scatter plot of age with hs-cTnT with only encounters where eGFR ≥ 90 mL/min/1.73m^2. Positive correlation. Each dot represents the median hs-cTnT value for all encounters with that age.

Supplemental Table 3. hs-cTnT values (in ng/L) at the 25th, 50th, 75th, and 99th percentiles stratified by sex, race, age group, CKD stage, history of atrial fibrillation, history of heart failure without removing outliers. Results are aggregated at the encounter level.

	n	***25th %tile***	***50th %tile***	***75th %tile***	***99th %tile***
**Gender**					
Female	5751	6	10	23	213
Male	3928	8	17	39	347
**Race**					
Black	8348	6	12	29	298
White	932	7	17	34	189
**Age Group**					
< 50	2951	6	6	11	235
50 – 64	3141	6	12	26	318
≥ 65	3587	12	22	43	267
**CKD Stage**					
No CKD	3391	6	6	12	85
Stage 2	3284	6	11	20	143
Stage 3	1864	14	24	40	212
Stage 4	419	29	48	84	370
Stage 5	125	44	68	114	324
ESRD	596	65	104	168	779
**Atrial Fibrillation**					
No	8611	6	11	26	262
Yes	1068	17	30	59	356

**Heart Failure**					
No	7378	6	9	20	212
Yes	2301	16	31	64	465

%tile, percentile

Supplemental Table 4. hs-cTnT values (in ng/L) at the 25th, 50th, 75th, and 99th percentiles stratified by multiple subgroups without outliers removed. Results are reported aggregated at the encounter level.

Gender	***Race***	***Age Group***	***eGFR***	***n***	***25th %tile***	***50th %tile***	***75th %tile***	***99th %tile***
Female	B	< 50	≥ 90	1045	6	6	6	36
			60 - 89	395	6	6	7	70
			< 60	212	12	30	87	433
Female	B	50 - 64	≥ 90	538	6	6	9	36
			60 - 89	591	6	7	12	103
			< 60	457	14	29	54	505
Female	B	≥ 65	≥ 90	260	8	13	22	92
			60 - 89	696	8	13	22	114
			< 60	892	15	28	54	272
Female	W	< 50	≥ 90	51	6	6	6	78
			60 - 89	48	6	6	6	62
			< 60	17	7	21	28	100
Female	W	50 - 64	≥ 90	17	6	10	18	55
			60 - 89	55	6	6	14	58
			< 60	31	10	19	32	134
Female	W	≥ 65	≥ 90	35	10	14	22	166
			60 - 89	98	9	16	28	272
			< 60	112	19	29	64	165
Male	B	< 50	≥ 90	516	6	6	10	91
			60 - 89	248	6	9	17	166
			< 60	163	36	74	154	741
Male	B	50 - 64	≥ 90	449	6	10	17	91
			60 - 89	414	9	14	24	214

			< 60	352	22	50	100	588
Male	B	≥ 65	≥ 90	227	11	17	27	82
			60 - 89	406	12	20	37	147
			< 60	487	28	50	99	452
Male	W	< 50	≥ 90	50	6	6	9	93
			60 - 89	33	6	8	19	148
			< 60	11	10	29	54	183
Male	W	50 - 64	≥ 90	32	9	14	20	403
			60 - 89	60	8	14	22	71
			< 60	39	25	48	65	511
Male	W	≥ 65	≥ 90	53	13	21	37	208
			60 - 89	79	12	20	29	98
			< 60	111	24	39	75	267

B, Black; W, White, %tile, percentile
